# Mechanical characteristics and energy evolution of rock with circular hole defects

**DOI:** 10.1371/journal.pone.0295675

**Published:** 2023-12-08

**Authors:** Zhongtang Xuan, Hongyan Li

**Affiliations:** 1 Chinese Institute of Coal Science, Beijing, China; 2 China University of Mining and Technology (Beijing), Beijing, China; 3 China Coal Research Institute, Beijing, China; Xi’an University of Science and Technology, CHINA

## Abstract

In this paper, the uniaxial compression damage characteristics of specimens are analyzed containing holes using PFC^2D^. In addition, the crack propagation, stress distribution and energy development characteristics of the specimens were systematically discussed. The findings indicate that the strength parameters of various specimens drop initially and then increase with increasing center point connecting angle, in comparison to intact rock. The most significant reduction in strength parameters is observed at a center point connecting angle of 45°. The stress concentration around the holes occurs prior to crack initiation and vanishes upon specimen failure. The number of cracks in the specimens is small and the propagation length is short before touching the peak value, while the cracks expand rapidly in a short period of time after the stress touches the peak value, and the crack development rules in the two stages are quite different. With increasing center point connecting angle, the pre-peak energy and total energy drop first and then increase. After touching the peak value, the specimen is dominated by energy release and the ability to absorb energy is weakened. In the case of center point connecting angle of 45°, the specimen has the largest energy difference coefficient and the worst ability to resist damage. The damage behavior can be composed of no damage, initial damage and accelerated damage take into account the damage change rules of the specimens.

## Introduction

Under the complicated geological action, there are numerous defects in the rock, which leads to the discontinuity and heterogeneity of the basic physical behavior of rock [1–5]. Additionally, it should be noted that defective rock typically exhibits lower strength and bearing capacity compared to intact rock. Among the numerous defect types, holes are more common. The mechanical and physical behavior of rock mass are significantly affected by the shape, size, and distribution of holes within the rock, as well as the properties of any fillers present [6–11]. As a result, understanding the impact of these factors on rock damage behavior is crucial for engineering projects.

Many scholars have shown great interest in the damage behavior of rocks involving various pore sizes. Gui et al. [12] adopted numerical analysis method to explore the damage behavior of rock with various pores size and confining pressure. Bai et al. [13] utilized discrete element method to demonstrate the impact of hole radius on the tensile strength and damage process of disc specimens. Huang and Yang [14] utilized PFC to conduct Brazilian splitting tests on disk specimens that had holes, and examined how the radius ratio of the hole to the disk affected the damage behavior and fracture propagation pattern of the specimens. The findings indicated that the presence of holes resulted in a notable decrease in the strength of the specimens. Zhao et al. [15] performed uniaxial studies on rock containing different hole diameters, and investigated the fracture initiation mechanism and the spatial distribution of cracks in combination with acoustic emission positioning system.

In addition, the relationships between the damage behavior of rock and hole quantity have also attracted the attention of many scholars. He et al. [16] investigated the impact of hole defects on the damage and energy transformation behavior of red sandstone through massive compression studies involving sandstone with varying numbers of holes. The findings revealed that the presence of holes had a notable impact on the damage behavior of sandstone. Zhou et al. [17] explored the damage behavior and crack propagation rules of rocks with different numbers of holes under impact loads using drop hammer impact and high-speed camera equipment. Huang et al. [18] utilized PFC^3D^ to perform uniaxial tests on granite samples with varying numbers of pores, aiming to examine how the number of pores affects rock strength and fracture behavior. Similarly, Sarfarazi et al. [19] employed PFC^3D^ to conduct Brazilian splitting tests on rocks with different hole quantities, revealing that the failure of disc specimens with holes was due to the propagation of radial tensile cracks. Wu et al. [20] prefabricated rock samples with varying numbers of horseshoe-shaped holes and investigated the impact of hole quantity on crack initiation and stress concentration coefficient around the holes based on the laboratory tests and numerical analysis methods.

However, the previous research primarily examines the changes in damage behavior of rocks based on varying hole sizes and quantities, but there has been limited investigation into the impact of hole distribution on the damage behavior of rocks. In fact, the holes in the rock mass are not singularly present, and there are other holes around the holes, which leading an inhomogeneous stress field in the rock mass. The presence of holes in rock formations can cause relative cracking and expansion when subjected to external loads, ultimately causing the loss of stability of rock slope. As such, it is crucial for both theoretical research and practical engineering to investigate how the distribution form of these holes impacts the damage behavior and fracture morphology of rock masses.

## Numerical model and microscopic parameters

**Fig 1:**
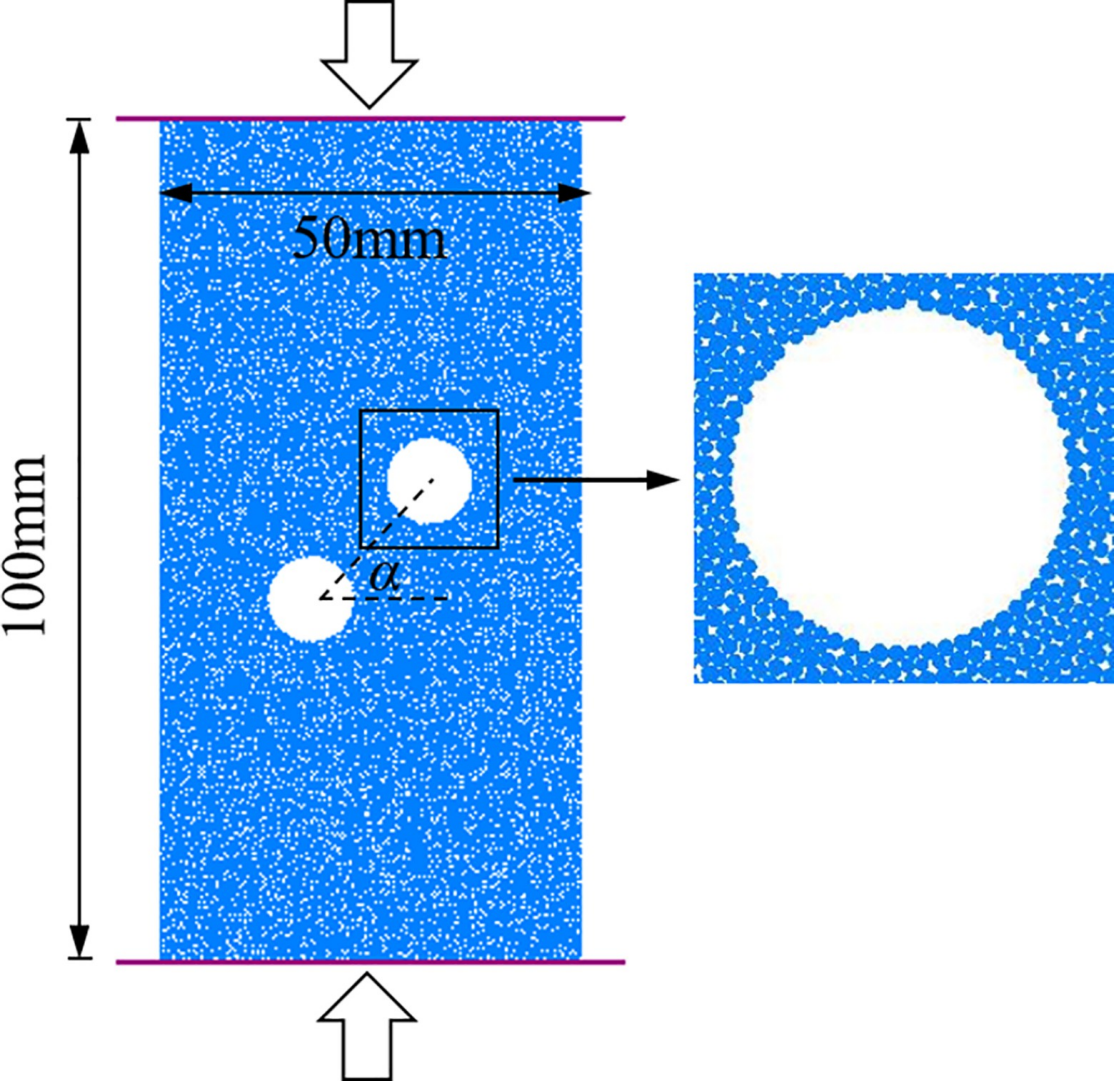
PFC^2D^ model for specimen.

In this paper, a computation model with holes was set up to reveal the damage behavior and energy variation rules of specimens under static conditions through PFC^2D^. In the model, the line connecting the center of the holes is an oblique line, and the angle between it and the horizontal line is the center point connecting angle, which is set from 0° to 90°. The numerical model has dimensions of 50 mm in width and 100 mm in height. The hole has a 10 mm diameter, and there are 20 mm between the central location of the holes. The numerical model is depicted in Fig 1. The setup of the microcosmic parameters is based on the result of [21]. The microscopic parameters of particles and contacts employed in this paper are shown in Table 1.

**Table 1:** Microcosmic parameters for the specimens.

Particle parameters	Values	Parallel Bond Parameters	Values
Minimum radius/mm	2.0	Young’s modulus/GPa	3.0
Ratio of maximum to minimum of radius	1.5	Ratio of normal to shear stiffness	1.5
Young’s modulus/GPa	3.0	Normal strength/MPa	45
Ratio of normal to shear stiffness	1.5	Shear strength/MPa	45
Friction coefficient	0.6	Radius coefficient	1.0
Density/(kg/m^3^)	2500		

## Results and analysis

### Mechanical characteristics

**Fig 2:**
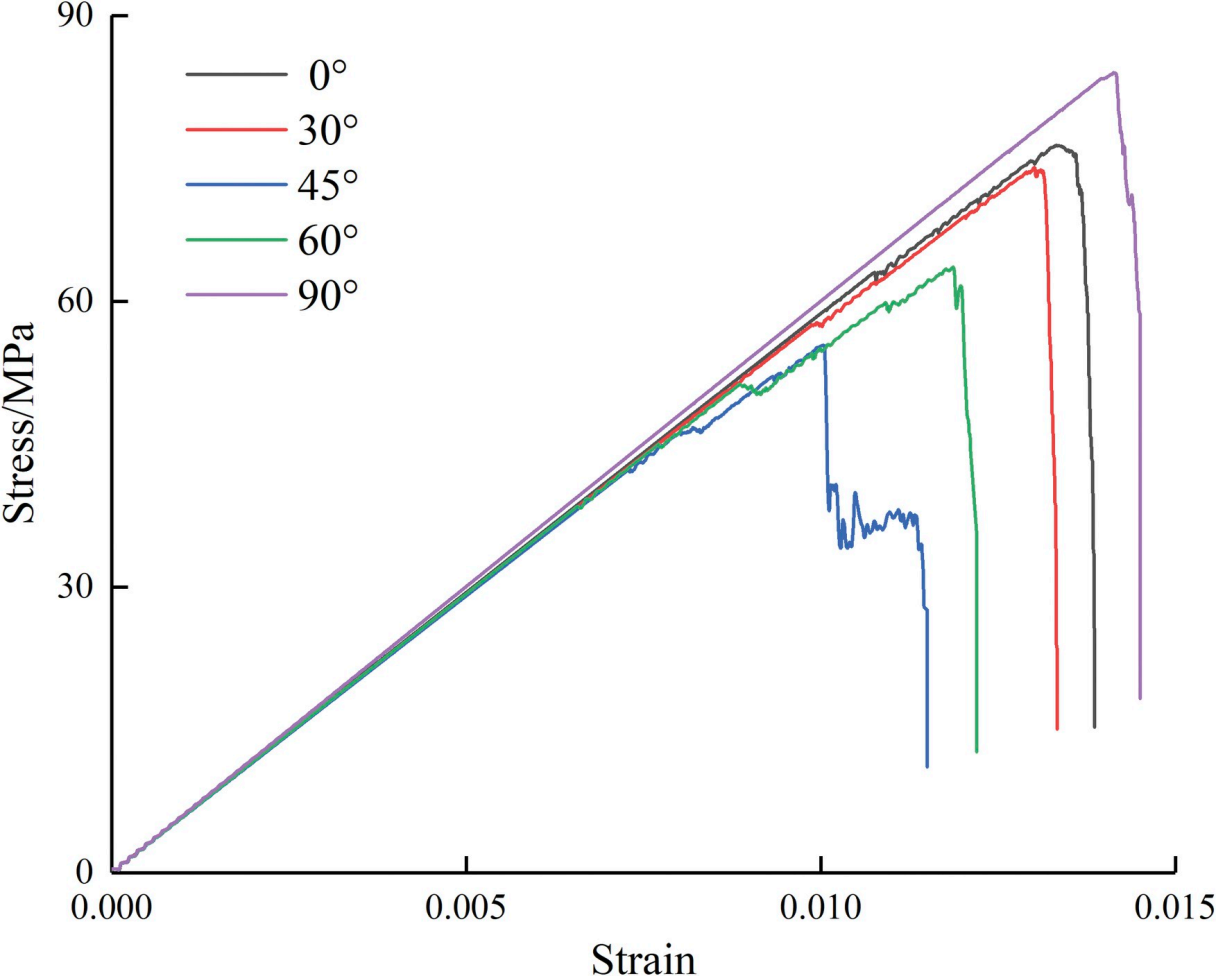
Stress-strain curves of specimens.

As shown in Fig 2, the stress-strain curves of the samples in different conditions are basically the same. At the early period of loading, the curves almost overlap together. Currently, the specimen is undergoing internal compaction, and the position of holes has minimal impact on the strength variations of the specimens. Prior to touching the peak value, the stress-strain curves exhibit nearly linear behavior. After touching the peak stress, the curves suddenly drops, and the stress weakens rapidly in a short period of time. The main reason is that after touching the peak value, the internal cracks of the samples extremely expand in a short period of time, which results in the specimen having a continuously decreasing load-bearing capacity. In the case of center point connecting angle of 45°, the stress-strain curve has an additional “plateau period”, which is strongly connected to the fracture extension rules and will be covered in more detail in the following.

**Fig 3:**
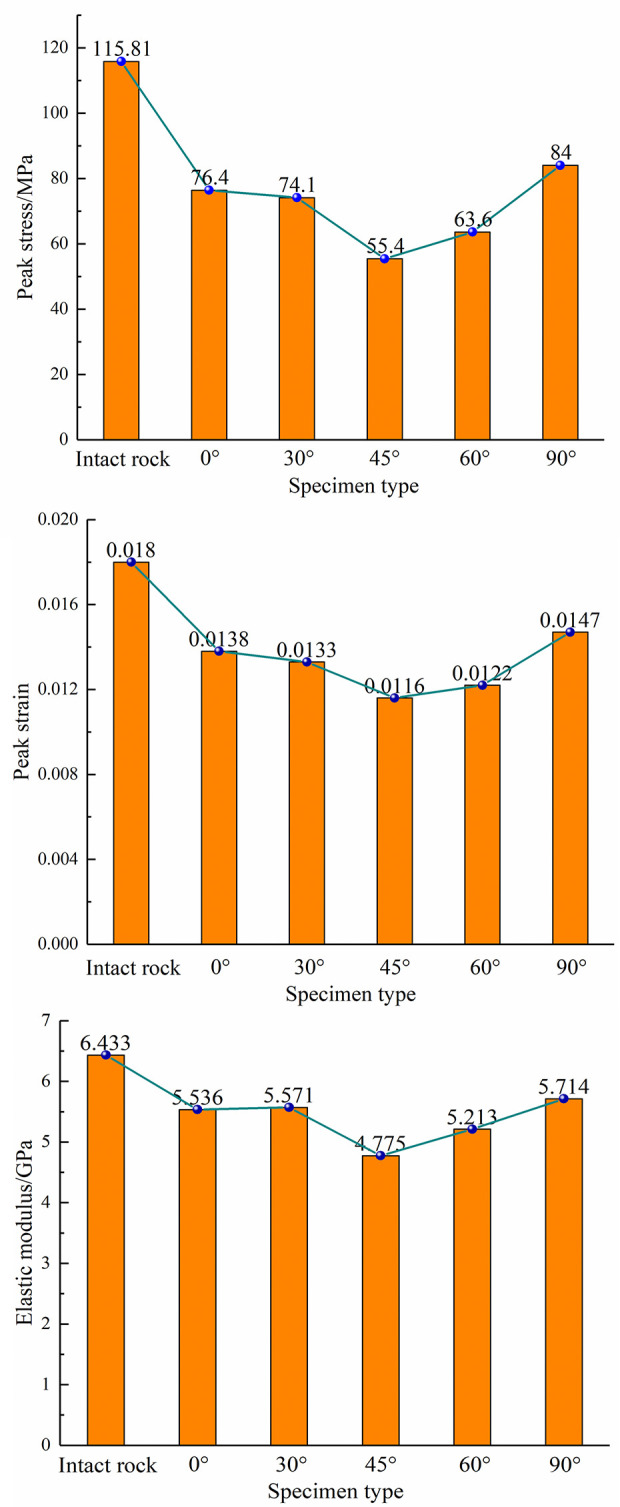
Variation curves of peak stress (a), peak strain (b) and elastic modulus (c).

According to Fig 3, the strength features of the rock involving holes are much lower than those of the rock that is complete, proving that the existence of holes greatly lowers the strength behavior. Different types of strength features are reduced to varying degrees as compared to unbroken rock, and the overall strength reduction first declines and then rises with increasing center point connecting angle. Among them, the largest decrease in peak intensity was observed. The greatest decrease in strength parameters was observed for a center point connecting angle of 45°, with 52.16%, 35.5%, and 26.1% for peak stress, peak strain, and elastic modulus, respectively. While the smallest decrease in strength parameters was observed for a center point connecting angle of 90°, with 27.46%, 18.33%, and 11.17% for the three strength parameters, respectively.

### Crack propagation and stress evolution process

**Fig 4:**
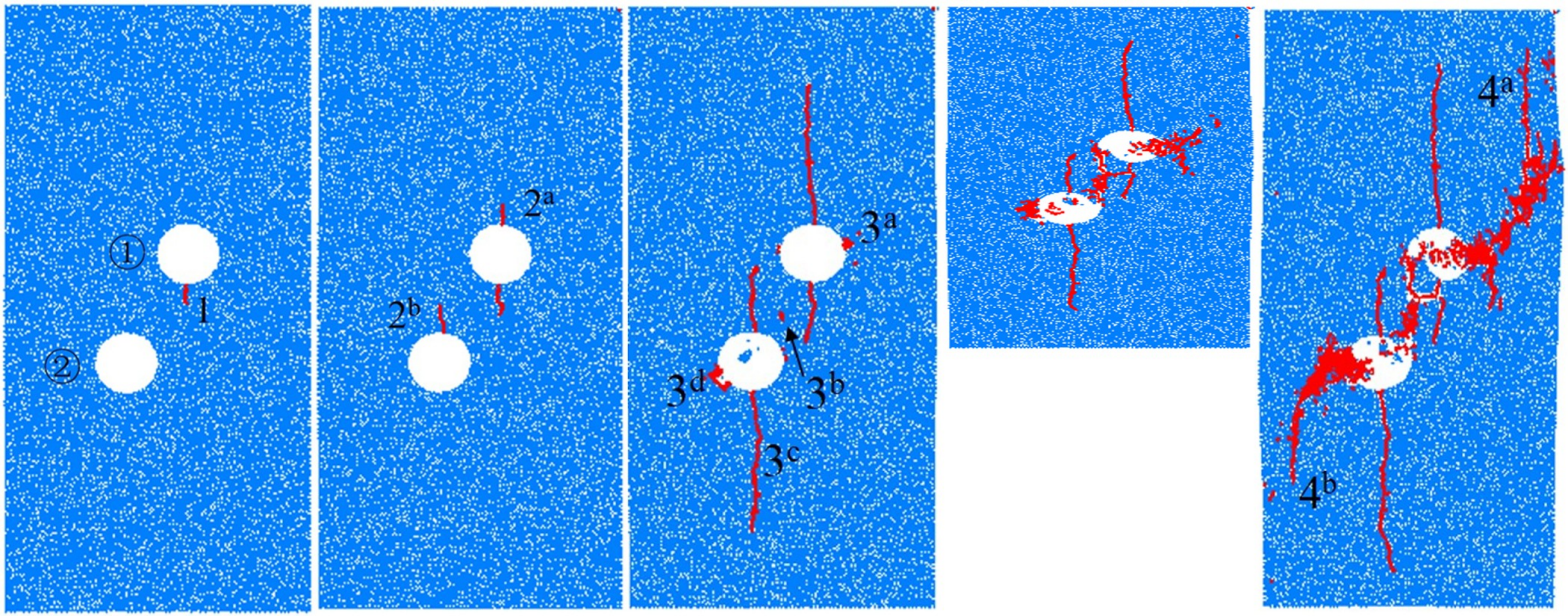
Crack propagation process of specimen. (a) *σ*_1_ = 38.5MPa (b) *σ*_1_ = 51.1MPa (c) *σ*_1_ = 63.6MPa (d) *σ*_1_ = 51.9MPa (e) *σ*_1_ = 31.9MPa.

The crack propagation of specimens at different moments can be obtained by PFC^2D^, which can clarify the crack extension and closure process of specimens with holes. In this paper, the specimen with a center point connecting angle of 60° is selected as a case to specifically analyze the crack extension and stress evolution rules. As shown in Fig 4, when the stress is loaded to 38.5 MPa, the crack occurs in the specimen for the first time, and crack 1 generates from the bottom side of hole ①. In the case of stress of 51.1 MPa, crack 2a and 2b initiate and propagate upward above hole ① and hole ② respectively, and the propagation length of crack 1 is small. At this time, the specimen is not macroscopically damaged, and the quantity of cracks is only 44. With increasing stress, in the case of stress of 63.6MPa, the amount of cracks grows to 302. At this time, the cracks 1, 2a, and 2b continue to vertically expand, and the propagation length is longer. Cracks 3a and 3d are generated on the outside of the holes, and secondary crack 3b initiates in the midsection of the holes, while crack 3c initiates below hole ② and extends downward for a long distance. When the stress touches 51.9 MPa, the four wing cracks do not change significantly compared with the case of 63.6MPa, while the crack 3b expands to the outside of the holes under load, and the hole closure process is completed. While cracks 3a and 3d only produce local damage around the holes under load. At this time, the extent of damage of the specimen is further aggravated, and the quantity of cracks grows to 721. When the stress touches 31.9 MPa, cracks 4a and 4b start from the local failure area on the outside of the holes and expand along the loading direction. At this time, the quantity of cracks reaches 1535, and the specimens have a high failure degree. The specimens are damaged and lost the load-bearing capacity.

**Fig 5:**
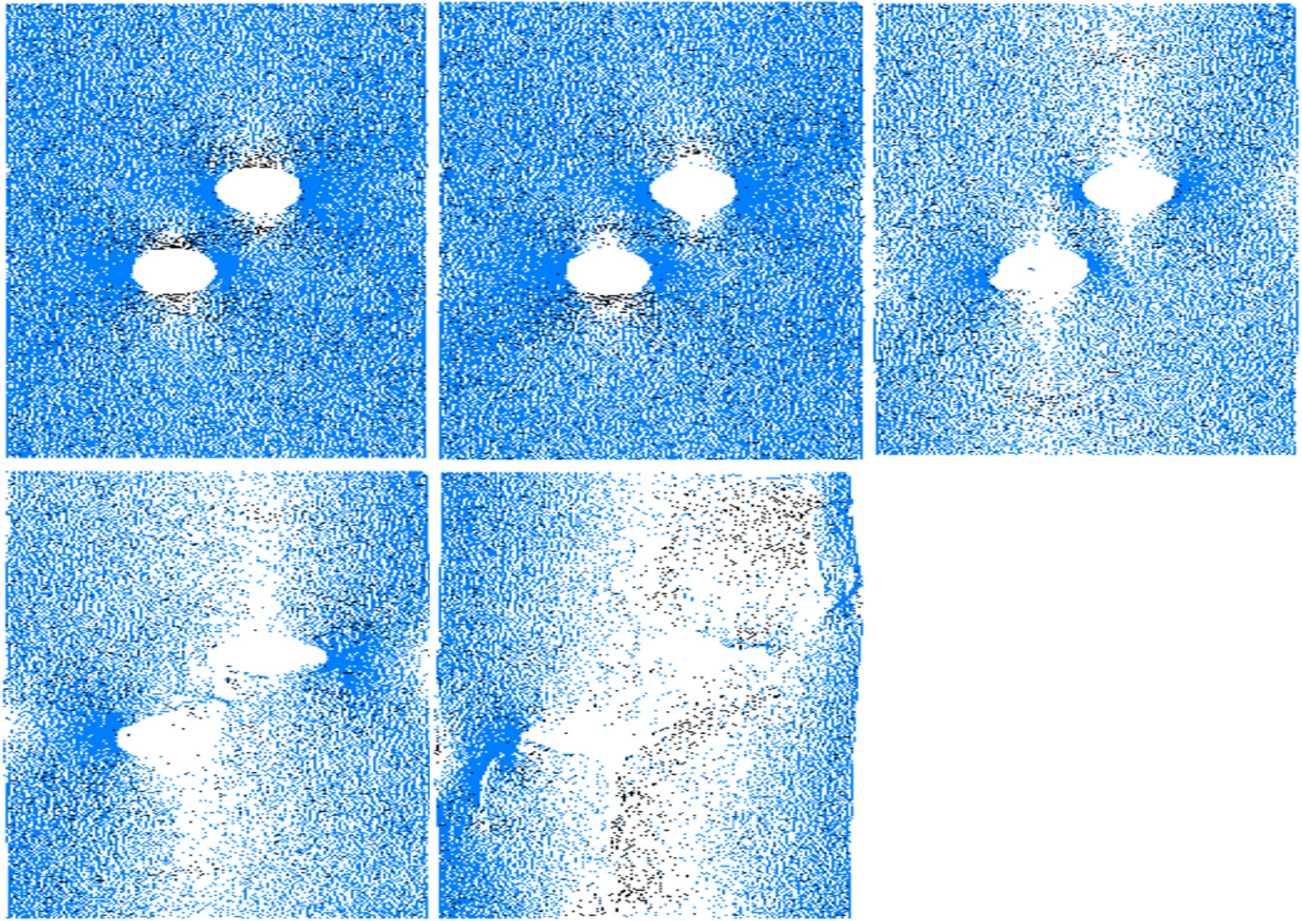
Contact force chain evolution process of specimen. (a) *σ*_1_ = 38.5MPa (b) *σ*_1_ = 51.1MPa (c) *σ*_1_ = 63.4MPa (d) *σ*_1_ = 51.9MPa (e) *σ*_1_ = 31.9MPa.

As shown in Fig 5, blue segments represent compressive stress and black segments represent tensile stress. In the case of stress of 38.5 MPa, stress concentrations are present around the holes, and the stress distribution shows no difference. In the case of stress of 51.1 MPa, the tensile stress around the holes weakens, and the stress in other parts does not change significantly, which indicates that the most probable driver for the crack generation is tensile stress. When the stress reaches 63.4 MPa, the tensile stress around the holes almost disappears, and the compressive stress around the holes and inside the specimen weakens, which indicates that the change of stress results in crack propagation. The midsection of the specimen displays a clear stress sparsity as the specimen enters the later period and the stress touches 51.9 MPa, and compressive stress concentration is mainly appears outside the holes. In the case of stress of 31.9 MPa, there is obvious tensile stress in the midsection of the specimen, and the compressive stress is dominant at the edge of the specimen, and the stress between the holes becomes very sparse. The fissure propagation rules depicted in Fig 5 and the stress evolution mechanism are mostly comparable. The stress at the position of crack propagation becomes sparse, and the fundamental cause for crack propagation is the tensile stress that occurs in fracture expansion.

### Force chain distribution

**Fig 6:**
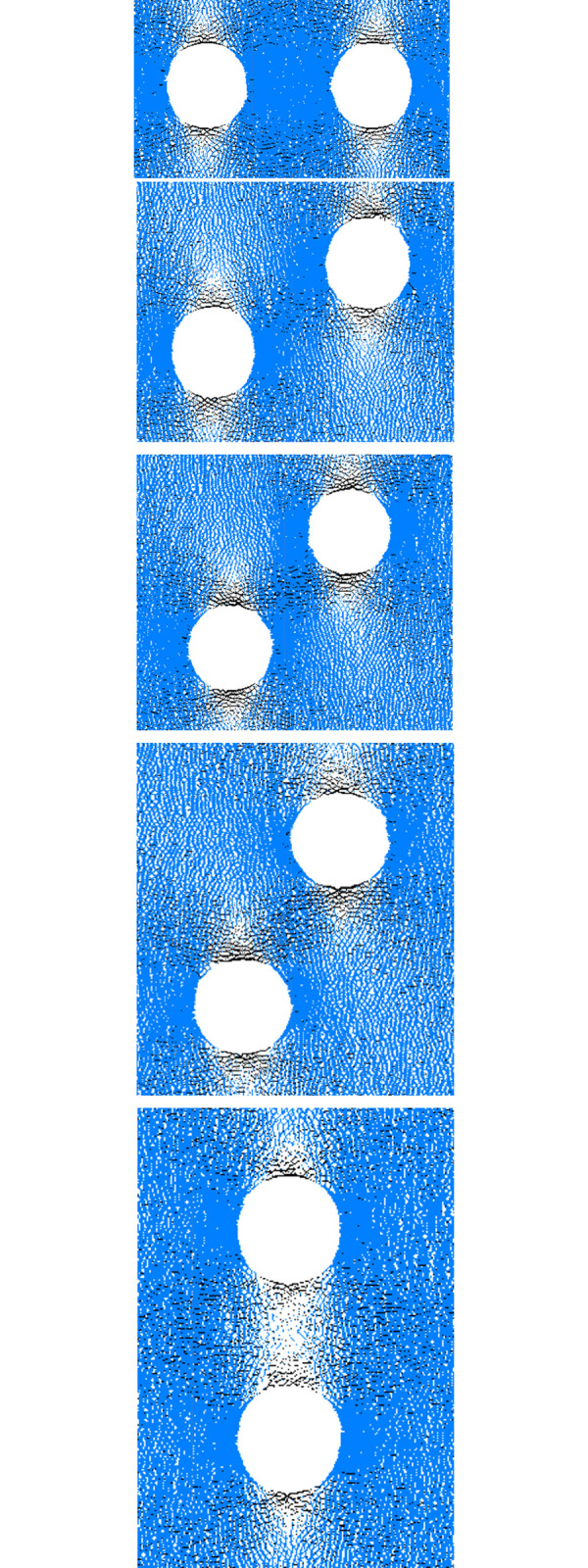
The distribution of contact force chain before cracking.

**Fig 7:**
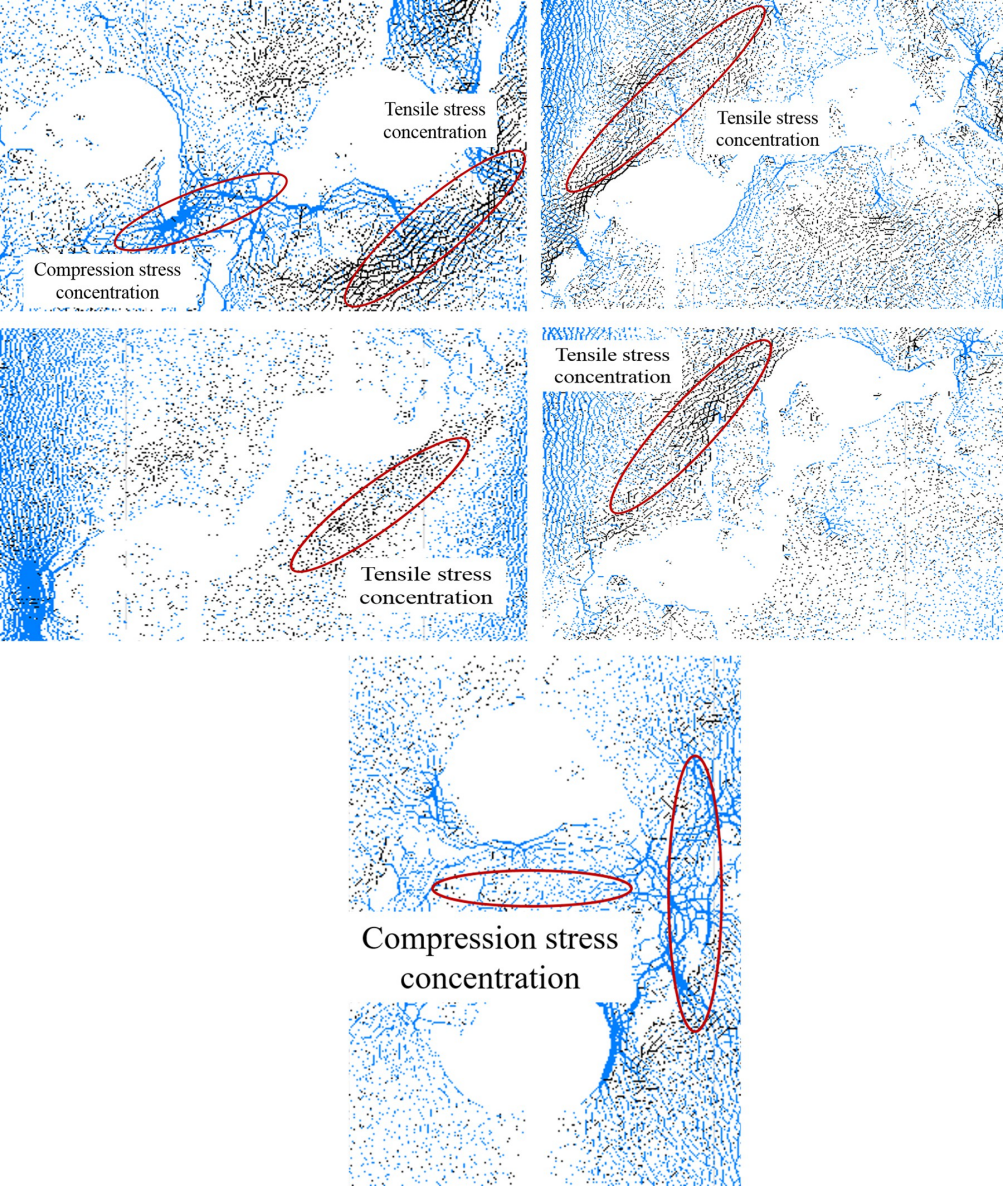
The distribution of contact force chain after cracking.

Understanding the force chain distribution is crucial in comprehending the mechanism of crack generation and extension, as it is closely linked to the propagation of cracks [22–27]. To illustrate the relationships between them, Figs 6 and 7 depicts the contact force chain distribution around the holes before and after damage, with blue indicating compressive stress, black indicating tensile stress, and the intensity of color representing the magnitude of stress. As depicted in Figs 6 and 7, prior to the initiation of cracking, the two holes exhibit tensile stress concentration on the upper and lower sides, while compressive stress concentration is observed on either side of the holes. Additionally, the rock bridge region between the holes experiences compressive stress concentration, which gradually diminishes with increasing center point connecting angles. When the stress is concentrated to a certain level, the cracks will be appeared.

When the specimens are destroyed, the force chain around the holes becomes sparse, which suggests that the stress at the corresponding location is weakening. The concentrated stress existing around the holes before crack initiation has basically disappeared, but the concentrated stress areas are observed at other positions after the specimen is destroyed, which demonstrates that the existence of holes has a notable impact on the change of the internal stress field of the specimens. When the specimen was damaged and the center point connecting angle reached 0°, there was a concentration of compressive stress between the holes, while the right and lower sides of the right hole exhibited some concentrated tensile stress areas. However, in the case of center point connecting angle of 30°, 45° and 60°, the concentrated tensile stress areas are observed on the outside of the hole, and its distribution is parallel to the direction of rock bridge. In the case of center point connecting angle of 90°, there are concentrated compressive stress areas in the middle and right sides of the holes.

### Crack initiation stress

**Fig 8:**
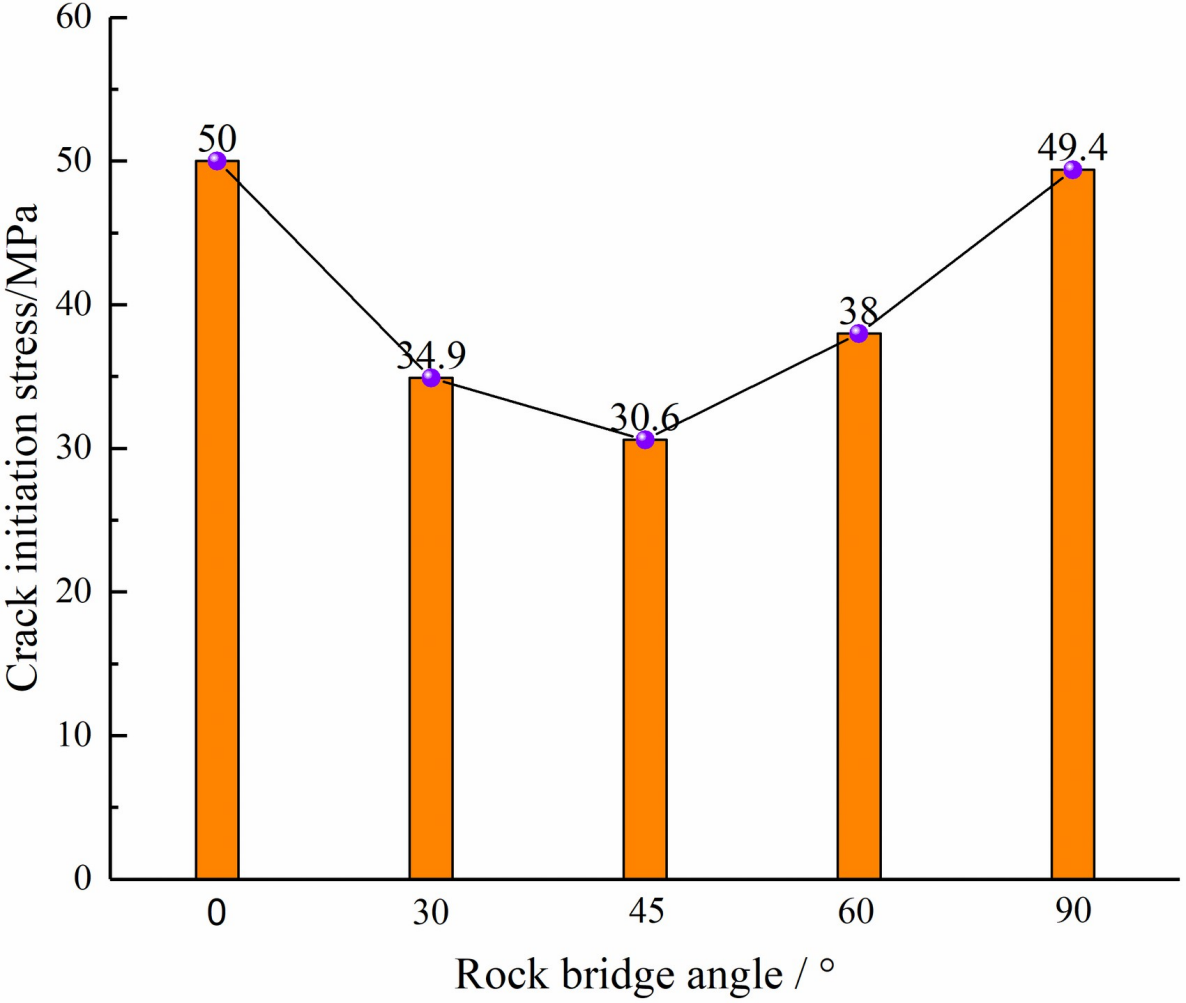
Variation curves of crack initiation stress.

As depicted in Fig 8, in accordance with the center point connecting angle, the crack initiation stress exhibits a ‘V’ shaped pattern. Specifically, the cracking stress gradually drops from 0° to 45° and then rises from 45° to 90°. Notably, the minimum cracking stress of 30.6 MPa exists at a center point connecting angle of 45°, while the maximum cracking stress of 50 MPa exists at a center point connecting angle of 0°. In the case of center point connecting angle of 30°, 45°, and 60°, there is a certain shear force between the holes due to the location of holes distribution, which accelerates the crack initiation process, leading to an earlier crack occurrence time and a lower crack initiation stress.

### Energy evolution

There is a close connection between crack initiation and energy evolution. During the loading period, energy absorption from the outside is continuously progressing inside the rock, which will be stored in the specimens. The energy will be discharged in huge amounts when reaching the storage limit, accelerating the damage process of the specimens [28, 29]. The amount, length and spatial arrangement of cracks significantly vary before and after touching the peak value, as shown in Figs 6 and 7, indicating that the energy development rules of the specimens in the two stages are not the same. Clarifying the energy development mechanism of rocks and the energy differences between the two stages is crucial for clarifying the crack propagation mechanism. In practical engineering, when the reinforcement of rock containing defects is required, a thorough comprehension of the relationship between energy and crack expansion is necessary to formulate an accurate reinforcement plan, achieve satisfactory reinforcement effects, and reach the purpose of preserving the stability of the rock structures. Under uniaxial stress, elastic and dissipative energy are the main components of energy absorption from the outside. The energy inside the rock can be calculated by Eq (1)–Eq (3) [30, 31].

$$U=\int_{0}^{\varepsilon_{u}}\sigma d\varepsilon$$ (1)

$$U^{e}=\frac{\sigma^{2}}{2E_{0}}$$ (2)

$$U^{d}=U-U^{e}$$ (3)

where *ε*_u_ is the limit strain, *E*_0_ is the initial elastic modulus, *U*, *U*_e_ and *U*_d_ are total strain energy, elastic strain energy and dissipated strain energy, respectively.

**Fig 9:**
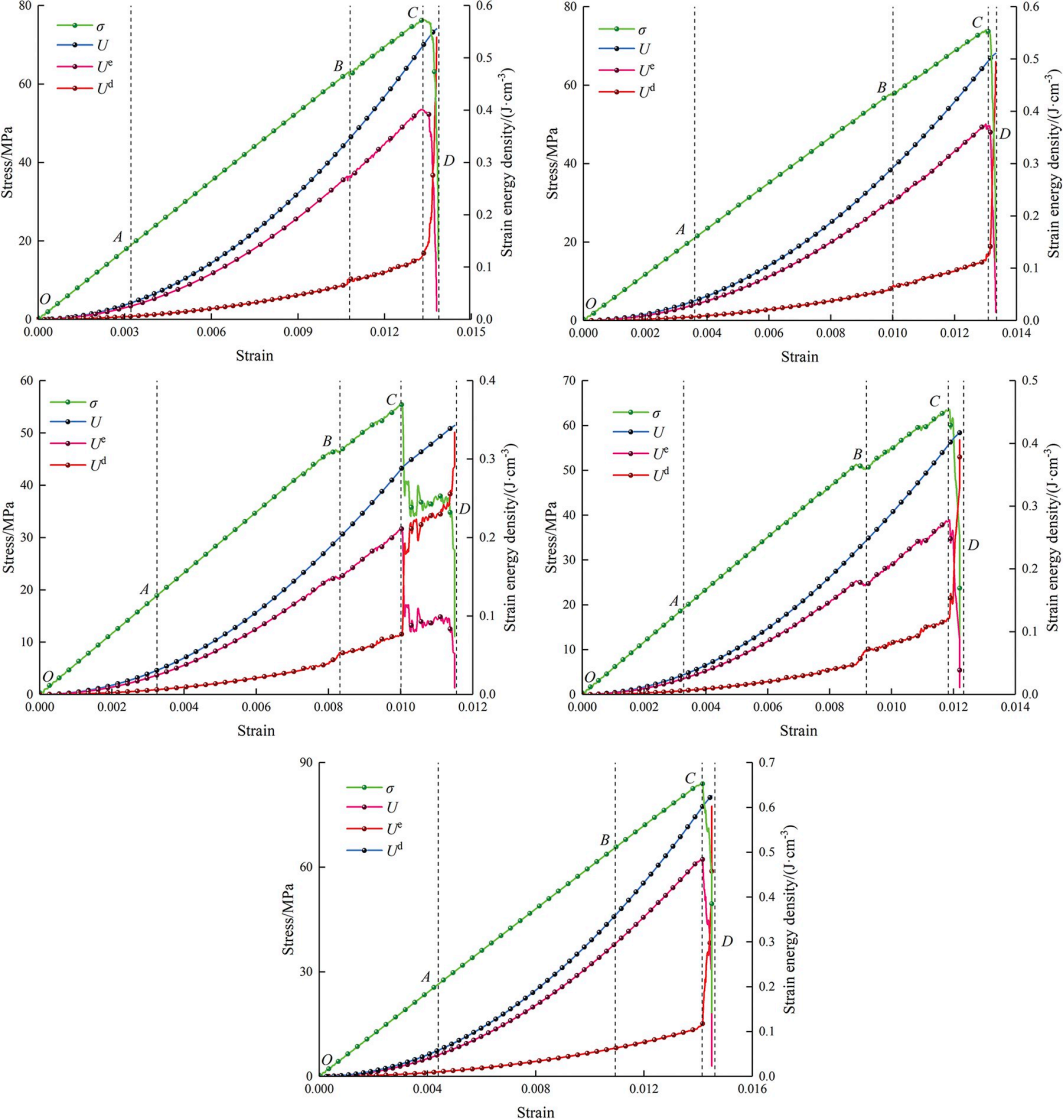
Variation curves of stress and energy evolution.

Fig 9 depicts the relationships of stress and energy evolution at different center point connecting angles. In the light of the energy variation curves and the damage behavior of the specimens, the energy evolution process can be composed of four parts, namely the energy quiet period (*OA*), the energy accumulation period (*AB*), the energy jumping period (*BC*) and the energy release period (*CD*).

#### The energy quiet period (*OA*)

At this stage, the specimens are deformed and gradually compacted under load. The pore structures are progressively closed, and the compacted space of the specimens increases. The total energy shows no difference with elastic energy, resulting in the dissipated energy is almost 0. In the energy quiet period, the specimens are mainly compacted, the specimen is not macroscopically damaged, and the energy consumed is almost 0. The energy in elastic form occupies the biggest amount of the energy absorption from the outside, and the specimens are dominated by energy storage.

#### The energy accumulation period (*AB*)

With increasing strain, the three types of strain energy continuously increase. The continuous increase of dissipated strain energy indicates that crack initiation and propagation occur inside the specimens, resulting in different damage degree of the specimens. The cracks consume a part of energy during the propagation process, but the energy in elastic form is much larger than that in dissipated form at this stage, suggesting that the specimens are still dominated by energy storage.

#### The energy jumping period (*BC*)

As the load increases, the three types of strain energy continue to increase. The specimen is not macroscopically damaged before touching the peak value, the crack propagation consumes less energy, and the specimens are still able to bear the loads. At this stage, the specimens are still dominated by energy storage. The more energy is stored, the less susceptible the specimens are to be damaged by energy driven.

#### The energy release period (*CD*)

The energy in elastic form drops drastically, but the energy in dissipative form grows drastically after touching the peak value. At this stage, it is hardly possible for the specimens to derive energy, the energy stored in the specimens is drastically released, the energy in dissipative form replaces the energy in elastic form, and the specimens are dominated by energy dissipation. In the case of stress of peak value, the internal cracks of the specimens keep expanding, the quantity and length of cracks continue to increase, resulting in increased damage degree and decreased load-bearing capacity inside the specimens.

**Fig 10:**
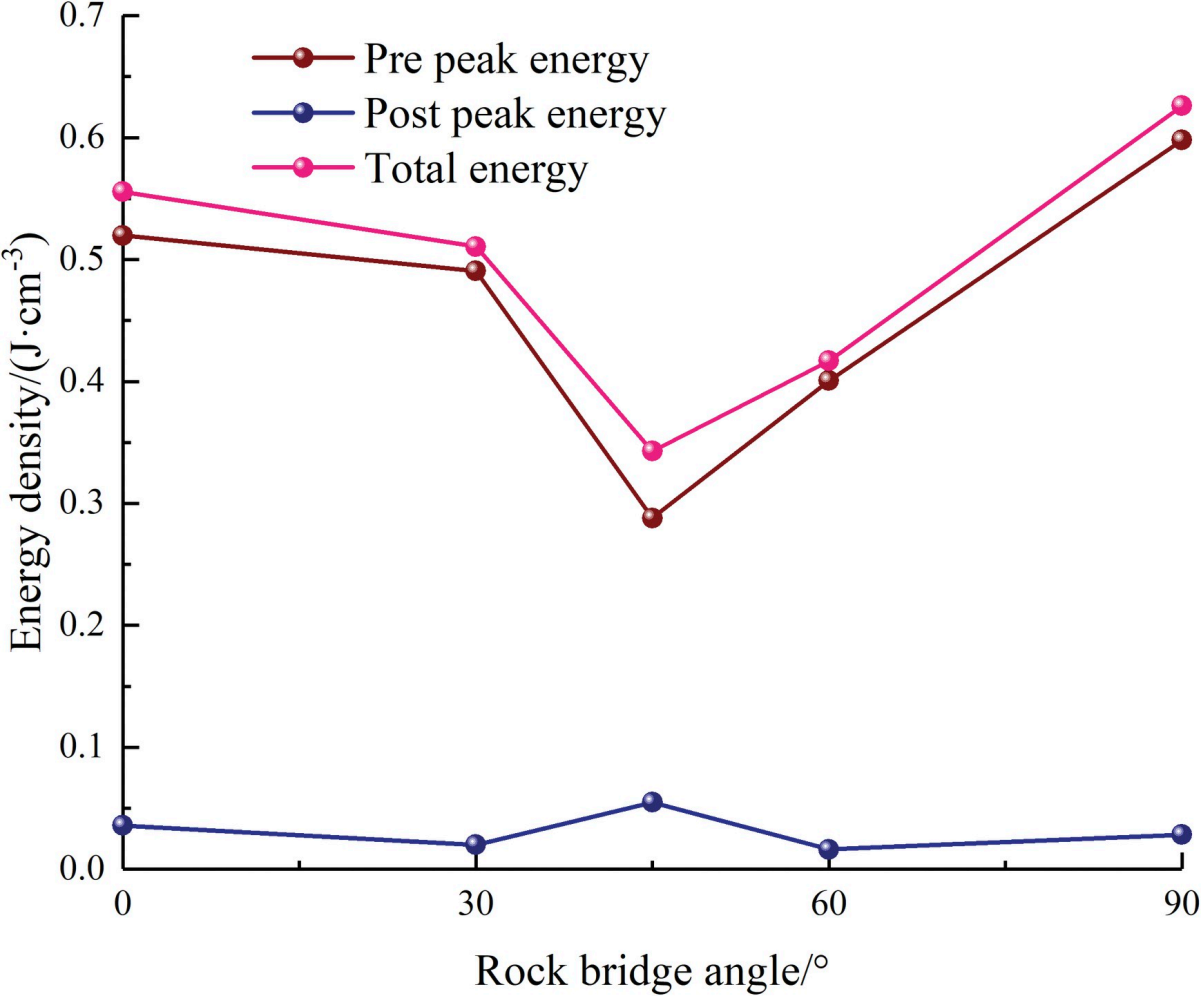
Variation curves of energy density.

As depicted in Fig 10, the energy absorbed by the specimens during the pre-peak stage initially drops and then rises with increasing center point connecting angle, with a small difference between the two energies. The pre-peak energy constitutes 84% to 96% of the total energy absorbed, while the post-peak energy is relatively low. The more energy absorbed before touching the peak value, the specimen is more resistant to damage and the crack is less likely to expand, which also leads to a huge quantity of energy being discharged from the specimen at the post-peak stage, prompting the cracks to expand and aggravating the damage of the rock. After touching the peak value, the specimen is dominated by energy release and the ability to absorb energy is diminished.

**Fig 11:**
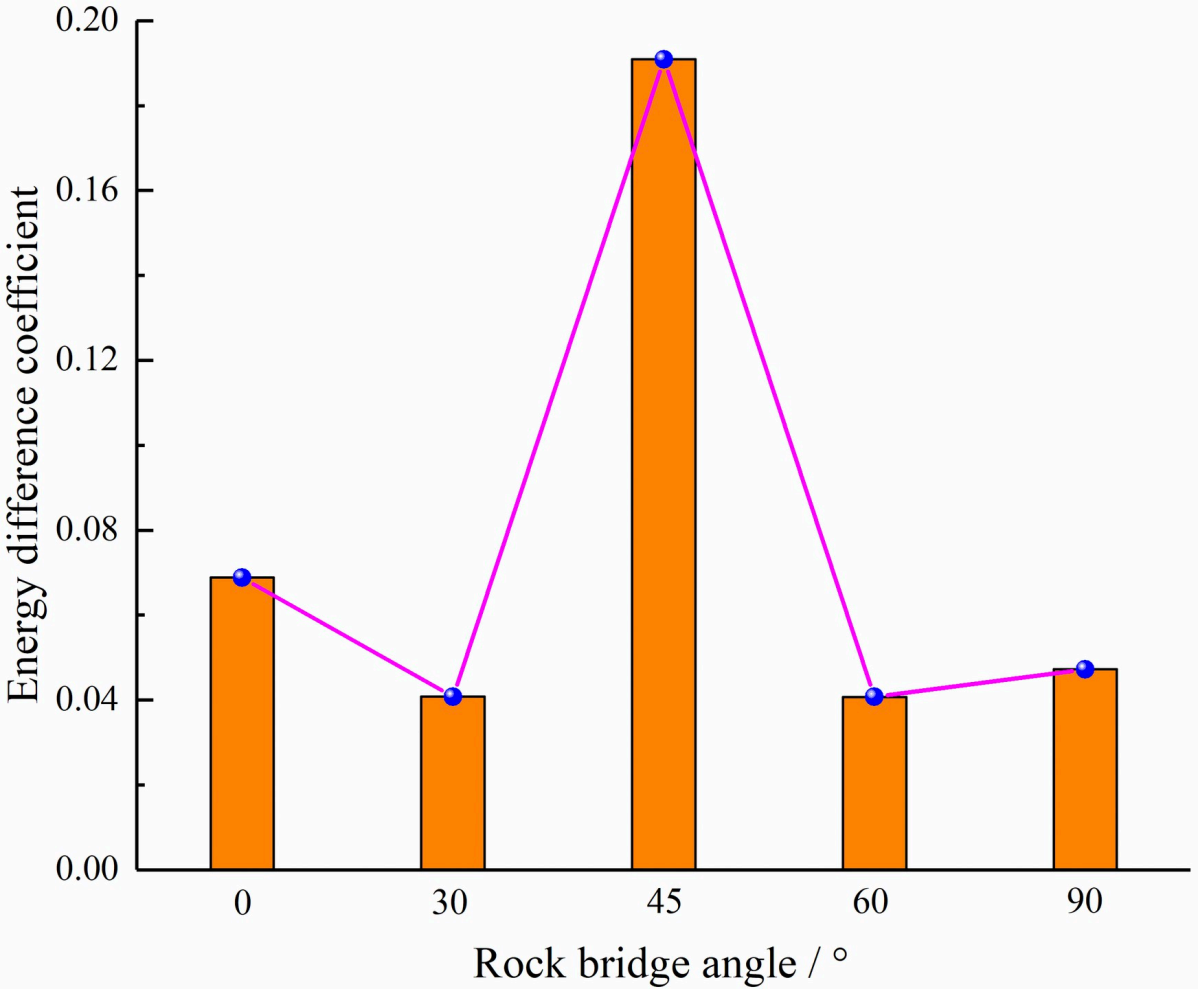
Variation curves of energy difference coefficient.

The energy difference coefficient is introduced in this study to measure the capacity of the specimen to withstand damage, which is determined by the ratio of post-peak energy to pre-peak energy. A higher value indicates a weaker resistance to damage. As depicted in Fig 11, in the case of center point connecting angle of 45°, the energy difference coefficient is higher than the other cases, indicating that the specimen in this case has the worst resistance to failure, which can also be confirmed by the stress-strain curve and the variation rule of peak strength. The specimen absorbs less energy before touching the peak value, so that the specimen does not suffer severe damage in a short period of time after touching the peak value. The ability to absorb energy after touching the peak value is higher than other situations. At this time, the specimen is still able to withstand the loads, which also leads to a “plateau period” in the stress-strain curve of the specimen after touching the peak value.

### Acoustic emission characteristics

In laboratory tests, acoustic emission technique has great advantages in monitoring crack propagation and spatial distribution in rock. In the PFC^2D^ model, similar principles can be used for acoustic emission analysis. Under compression conditions, the rock is not damaged at once to complete, often the process of gradual damage. In the parallel bond model, with increasing stress, when the force exceeds the bond strength between particles, microcracks will be generated between the particles, and the formation of a micro-crack corresponds to the quantity of events. The above principle allows the quantity of cracks to be monitored, and the maximum quantity of cracks at intervals is the quantity of acoustic emission events. Many scholars have conducted research to confirm that the results of acoustic emission analysis by PFC^2D^ show no difference with laboratory results [32, 33].

**Fig 12:**
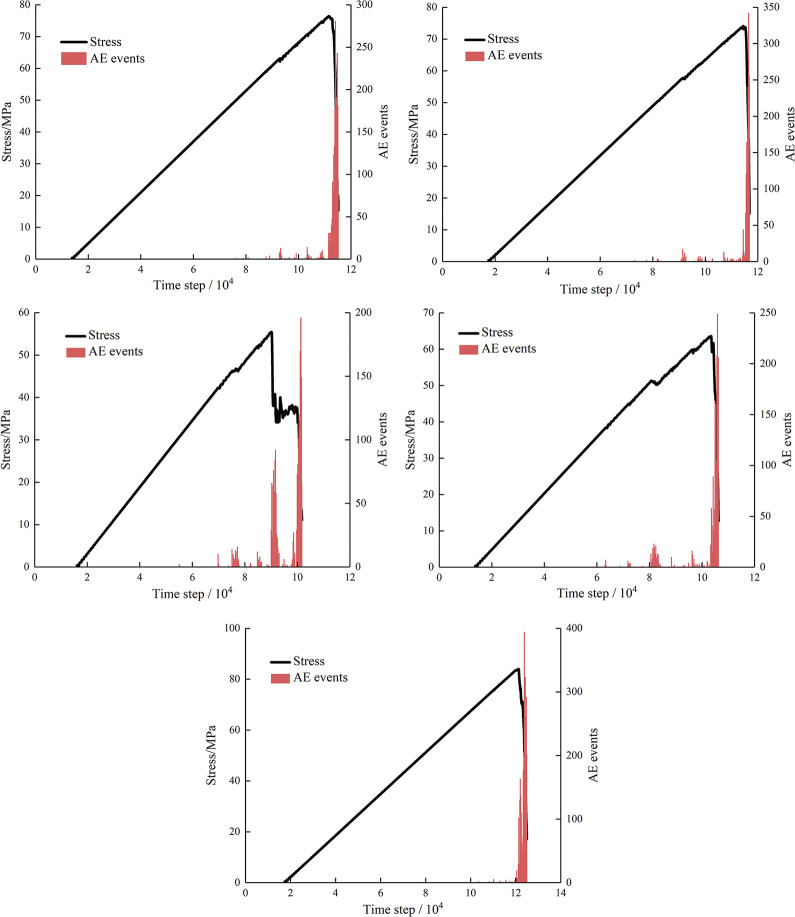
Variation curves of stress and AE events with time step.

As shown in Fig 12, when the stress declines, it corresponds to a large augment in the quantity of AE events. At the initial stage of loading, AE events was not measured, which shows difference from the indoor tests. This is because the specimens used in the indoor tests contain many microdefects, which will produce different degrees of microcracks propagation under uniaxial compression. The microcracks were not set in the numerical model established by PFC^2D^, and there was no microcracks extension inside the specimens at the preliminary stage, resulting in no corresponding AE events recorded. As the increase of stress, there are several small stress drops before the case of stress of peak value, indicating that micro-cracks propagation exists in the specimens, and the corresponding AE events is recorded. The greater the stress drop is, the more the quantity of AE events. When loaded to the peak value, the stress decreases rapidly, the microcracks inside the specimens expand in large quantities, and the quantity of AE incidents dramatically rises. Then the specimens are not able to bear the loads at all, and the acoustic emission stops.

### Damage evolution law

Under compression conditions, cracks generate and expand in the rock structures, which will affect the damage behavior of rock. With the expansion of cracks and redistribution of stress, the rock structures will possess a growing damage degree. Therefore, it is meaningful to set up an appropriate damage model to reveal the damage behavior of the rock structures. What is more, it is possible to formulate the engineering reinforcement plans according to the damage behavior to ensure the safety and normal development of the projects.

Assuming that the accumulated AE ringing times is *N* when the non-destructive section area *A* is entirely unable to bear the loads, and the accumulated ringing times is *N*_m_ when the section damage area is *A*_m_, the damage variable *D* can be stated by the following equation [34]:

$$D=\frac{N_{m}}{N}$$ (4)

Since rock specimens are hardly possible to be completely destroyed through PFC simulation analysis, the damage correction variable *D*_u_ is introduced in this paper, and the correction formula is as follows [35]:

$$D_{u}=(1-\frac{\sigma_{c}}{\sigma_{p}})\frac{N_{m}}{N}$$ (5)

where *σ*_c_ is residual stress, *σ*_p_ is peak stress.

On the basic of the strain equivalence theory and AE features [36], the damage constitutive model of specimens with double holes under compression conditions is established as follows [37]:

$$\sigma =E\varepsilon (1-D_{u})=E\varepsilon [1-(1-\frac{\sigma_{c}}{\sigma_{p}})\frac{N_{m}}{N}]$$ (6)

**Fig 13:**
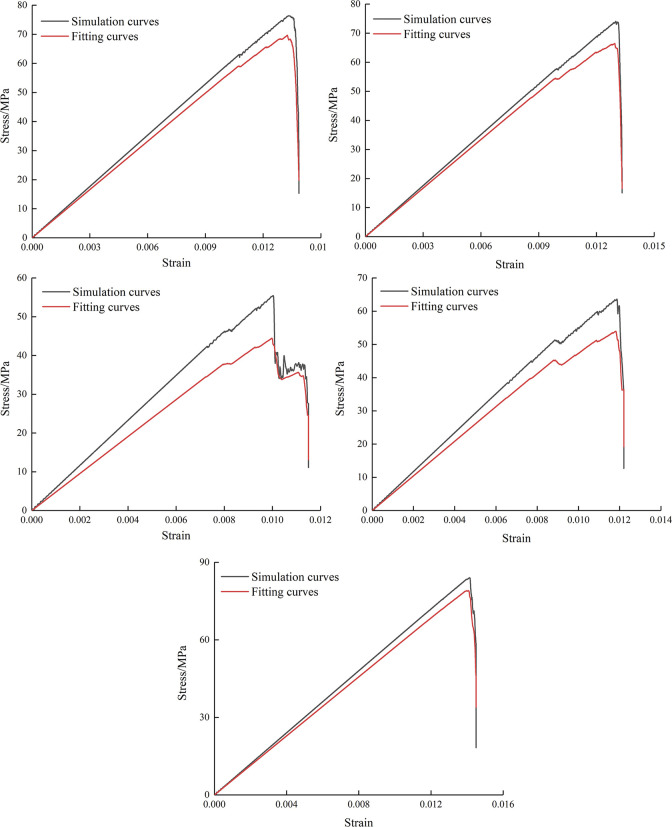
Fitting curves of specimens.

Fig 13 presents the simulation curves and theoretical curves of specimens derived by constitutive equation. As shown in Fig 13, the simulation curves at compression conditions are essentially consistent with the theoretical curves of specimens derived by constitutive equation, which indicates that the established model can correctly represent the damage behavior of the specimens.

**Fig 14:**
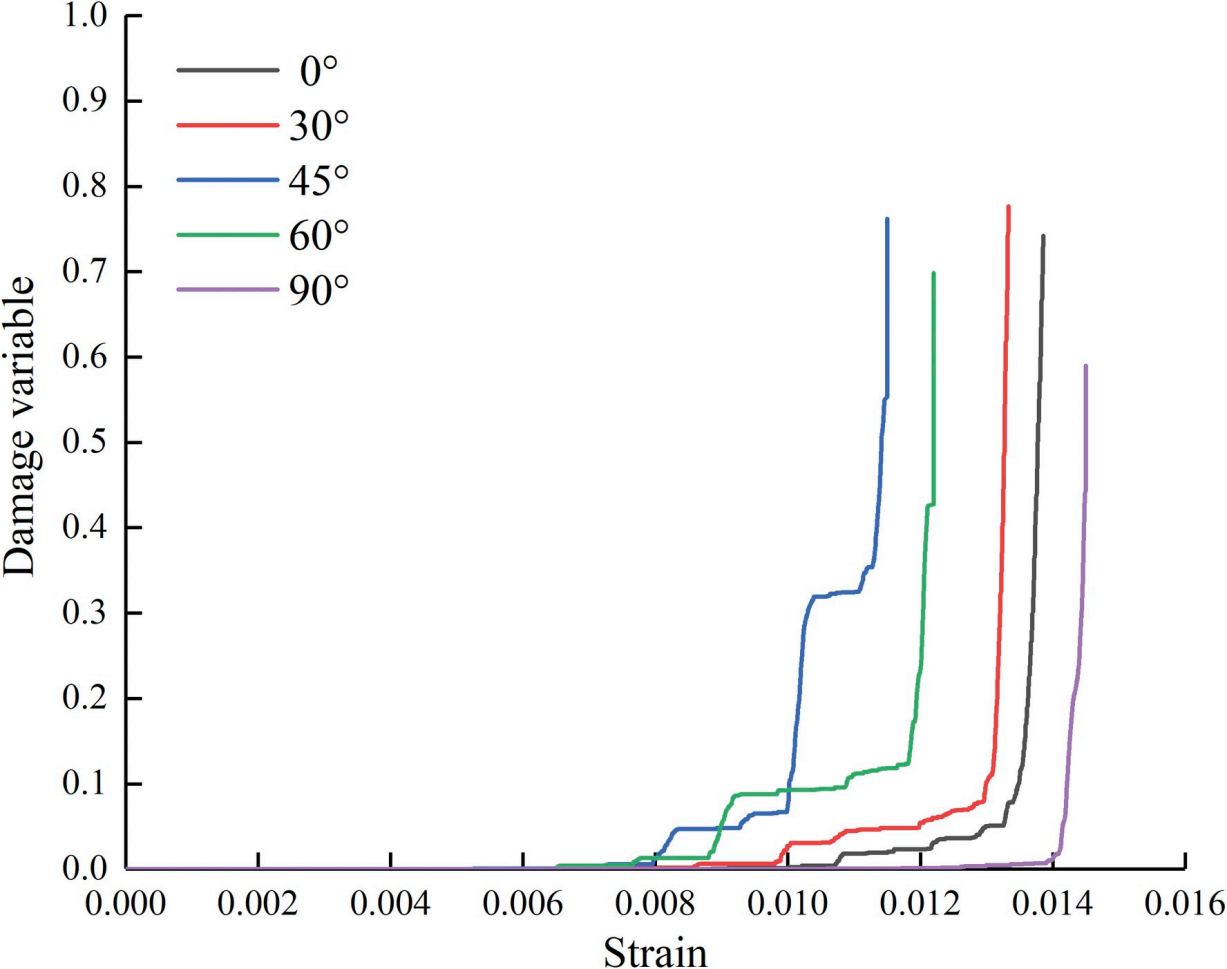
Strain-damage relationship curves of specimens with two holes.

Fig 14 presents the relationships between the center point connecting angles and strain-damage of the specimens. Take into account the rules of damage variable curves, the damage evolution can be composed of three parts: no damage, initial damage and accelerated damage. In the no damage stage, the specimens are deformed and gradually compacted under load. The pore structures are progressively closed, and the compacted space of the specimens increases. The internal stress of the specimen is small and there is no damage. In the initial damage stage, cracks are generated and continuously propagate inside the specimens, but the specimens still have high load-bearing capacity and the cracks expand slowly at this stage, resulting in a lower damage degree to the specimen. In the accelerated damage stage, the internal cracks of the specimens expand and close rapidly, and macroscopic damage locally occurs. The quantity of cracks drastically rises, the specimens have a high failure degree and finally are not able to bear the loads.

### Stochastic analysis

When building a PFC numerical model, it is necessary to use relevant commands to generate particles. However, the distribution of particles needs to be controlled by setting the seed number, otherwise the models will be inconsistent. The distribution of particles varies with the number of seeds, which has an influential effect on the damage response of the model. To ensure the precision of the numerical parameters and analysis results, it is imperative to investigate the mechanical response under various seed numbers, although the current study employs a seed number of 1e^4^. Therefore, the mechanical parameters of the model when the seed number is 2e^4^ and 3e^4^ are further explored in this paper, and compares with the results when the seed number is 1e^4^ to verify that the parameters set in this paper are highly reliable.

**Fig 15:**
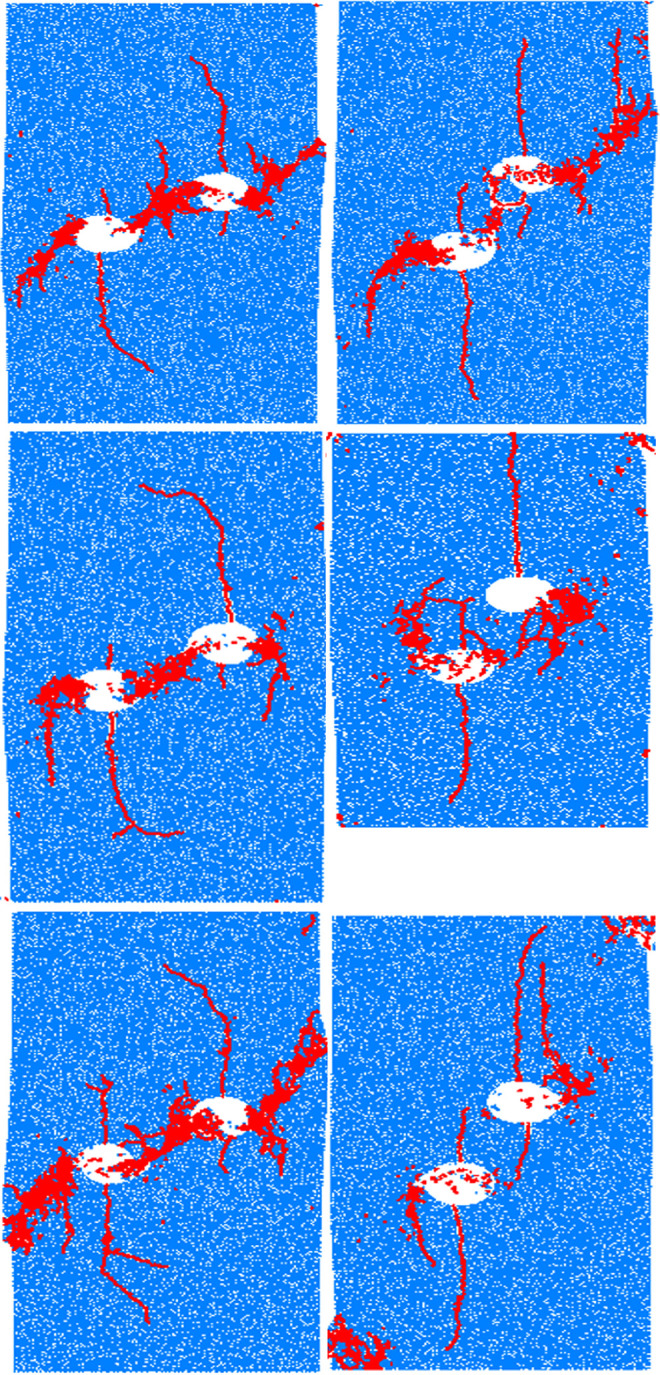
Failure modes with rock bridge gngle of 30° and 60°.

In this paper, the numerical models with center point connecting angles of 30° and 60° are chosen for different seed number analysis, where the mechanical parameters of the models at various conditions are displayed in Table 2. The difference between the two types of strength parameters of the models at different seed numbers is small and the error is within 5%. The high degree of consistency in the damage patterns of models with varying seed numbers is demonstrated in Fig 15. This observation serves as confirmation of the accuracy of the model parameters and analysis results that were utilized in this paper.

**Table 2:** Contrast of strength parameters at different random seeds.

Center point connecting angle	1e^4^		2e^4^		3e^4^	
	*σ*/MPa	*E*/GPa	*σ*/MPa	*E*/GPa	*σ*/MPa	*E*/GPa
30°	74.1	5.57	75.8	5.60	73.52	5.49
60°	63.6	5.21	65.28	5.24	66.5	5.27

## Conclusions

1. Compared to intact rock, the strength parameters of different types of specimens show a certain degree of reduction. The greatest decrease in strength parameters is observed for a center point connecting angle of 45°, with 52.16%, 35.5% and 26.1% for peak stress, peak strain, and elastic modulus, respectively. While the smallest decrease in strength parameters was observed for a center point connecting angle of 90°, with 27.46%, 18.33% and 11.17% for the three strength parameters, respectively.
2. The stress concentration around the holes is the primary cause of crack generation. The minimum stress value at which crack generation occurs is 30.6 MPa in the case of center point connecting angle of 45°, whereas the highest stress level reaches 50 MPa in the case of center point connecting angle of 0°.
3. With increasing center point connecting angle, the pre-peak energy and total energy exhibit a first decrease and then increase, and the pre-peak energy makes up 84% to 96% of the total energy. The specimens are primarily characterized by energy absorption before touching the peak value and energy release after touching the peak value. The energy difference coefficient reaches its maximum value in the case of center point connecting angle of 45°, indicating that the specimen exhibits the lowest resistance to failure.
4. The greater the stress drop, the more the quantity of AE events. With increasing stress, there is a slight stress drop before reaching the peak stress, indicating that there is microcrack propagation inside the specimens. The stress decreases rapidly when touched the peak value, numerous micro-cracks inside the specimens extend, the quantity of AE events drastically rise.

## Supporting information

S1 File Contains all data. (XLSX)

S2 File (ZIP)
